# Single incision thenar muscle reconstruction using the free functional pronator quadratus flap

**DOI:** 10.1186/s12893-021-01308-x

**Published:** 2021-07-12

**Authors:** Martin Aman, Arne H. Boecker, Mirjam Thielen, Camillo T. Mueller, Amir K. Bigdeli, Ulrich Kneser, Leila Harhaus

**Affiliations:** Department of Hand-, Plastic and Reconstructive Surgery, Burn Center, BG Trauma Center Ludwigshafen, Department of Hand- and Plastic Surgery, University of Heidelberg, Ludwig-Guttmann-Str. 13, 67071 Ludwigshafen, Germany

**Keywords:** Reconstructive surgery, Reconstructive microsurgery, Flap surgery, Plastic surgery, Free functional muscle transfer, Thenar muscle loss, Thenar muscle reconstruction, Thumb opposition

## Abstract

**Background:**

Injuries to the thenar muscle mass or the thenar branch of the median nerve and resulting loss of thumb opposition lead to a massive impairment of hand function. For decades, reconstructive approaches were based on tendon transfers. To broaden the reconstructive repertoire, we present the free functional pronator quadratus flap as a viable alternative for functional reconstruction and provide a specification for its indication. We demonstrate our surgical technique to a single incision reconstruction using the free functional pronator quadratus flap. Based on a series of three patients, which were analyzed for hand function using Kapandji’s score and the angle of Bourrel, grip strength and nerve conduction velocity in a two year follow up, we present an indication algorithm.

**Results:**

After successful reinnervation of all flaps, we found an improvement of Kapandji’s score from 4.3 ± 0.94 preoperatively, to 8.7 ± 0.47 after two years. Accordingly, the angle of Bourrel decreased from 75.75 ± 3.45 degrees to 36.96 ± 3.68 degree. Grip strength also improved from 14 ± 2.2 kg to 26.2 ± 1.2 kg. No impairment of wrist pronation was observed.

**Conclusion:**

We found excellent functional recovery of thumb opposition and strength, showing similar or even superior results compared to results from tendon transfers. With the benefit of a single incision surgery and therefore minimal donor site morbidity, this free functional muscle transfer is a viable alternative to classic tendon transfers.

**Supplementary Information:**

The online version contains supplementary material available at 10.1186/s12893-021-01308-x.

## Background

Loss of thenar muscle function is a rare, but considerable injury. Thus, this rather small trauma often leads to severe impairment of hand function considering range of motion, grasp function, and grip strength during occupational and daily life activities.

Since their first description in 1917, reconstructive approaches in hand surgery to restore thumb opposition are based on tendon transfers, accompanied with all their concomitant factors such as donor site morbidity and limited axes of movement [1, 2].

With the improvement of microsurgery, reconstructive approaches such as nerve transfers and free functional muscle transfers (FFMT) have become a viable option for functional reconstruction. Hereby, several case reports exist, describing FFMT for thenar reconstruction, including the gracilis flap, the serratus flap or the minor pectoral muscle flap [3, 4]. These flaps are all viable yet bulky options, but also come with the burden of a certain donor site morbidity.

The pronator quadratus (PQ) muscle flap was first described by Dellon et al. [5] as a small muscle flap with minimal donor site morbidity. Since it’s first description in 1983, it was only reported twice as a free functional flap case report of a single thumb reconstruction by Lee and Idler [6] and for facial reconstruction by Tzou and Aszmann [7], indicating the broad indication spectrum of this flap. With many potential benefits for thenar reconstruction, such as minimal donor site morbidity and excellent muscle properties it is a viable option for reconstruction [8, 9].

Therefore, we present a reconstructive concept for thenar reconstruction of thumb opposition using a free functional pronator quadratus flap (FFPQ). Furthermore, we present the outcome of a two-year follow up of our patients who underwent this functional reconstruction.

## Methods

Three patients (one female, two males) with a mean age of 38.6 years (range from 16 to 53 years) all suffering from a loss of thumb opposition underwent FFMT reconstruction using the ipsilateral PQ. All patients had a severe combined traumatic injury of the motor branch and thenar muscle mass and were transferred to our clinic after primary reconstruction efforts failed.

All patients were evaluated in a long-term follow up during routine examination for pre- and postoperative force as well as functional thumb opposition using Kapandji’s score and the angle of Bourrel [10–12]. This angle refers to the angle between the thumb and the fourth digit during opposition. Force was measured on both hands using a JAMAR dynamometer pre and postoperatively. Furthermore, neurological diagnosis was performed to assess the conduction velocity of the nerve as well as EMG of the thenar muscle to verify nerve damage and consecutive regeneration. Local institutional ethic consent was obtained and the principles according to the declaration of Helsinki were fulfilled.

### Reconstructive approach- preparation and patient selection

Admitted patients were examined for patient history followed by a detailed clinical examination to verify damage of the thenar branch and thenar muscles and to document preoperative hand function.

Patient one was a young (16y) student with a traumatic thenar laceration wound. Exploration of the wound showed severe loss of thenar muscle mass especially at the motor entry point of the thenar branch. Therefore, primary nerve repair was not feasible and a FFMT was performed immediately as this young patient and his parents demanded for optimum functional outcome. The second patient was a 46-year-old lawyer with a deep cut at the thenar, who received primary wound closure without further exploration in an emergency department. She was then referred with delay of almost a year after the injury to our center due to severe thenar atrophy and functional impairment. Intraoperative exploration then demonstrated complete injury to the thenar branch without potential for nerve transfer surgery due to prolonged and severe thenar muscle damage.

Our third patient (53y) had a work-related injury to the hand with combined muscle and nerve damage of the thenar region. A primary attempt of direct nerve repair was performed but was found to be insufficient in a one year follow up. After intraoperative exploration and preoperative discussion with the patient he wanted to undergo FFMT and denied tendon transfers due to potential donor site morbidity. We performed electrophysiological testing of nerve and muscle function of median nerve and thenar muscle electromyography in all patients. Neuroimaging with ultrasound further supported preoperative planning and helped with decision making for use of the PQ-flap. However, the preoperative workup in some cases cannot give the full information of the real quality of the functional structures. In these cases, the surgery should start with exploration of the situs and the surgeon has to be prepared for performing any other technique of the armamentarium of thenar reconstruction, such as tendon transfers or nerve transfers.

### Surgical procedure of the free functional PQ

A typical skin incision for open carpal tunnel release is made and extended to the forearm in line of the modified Henry approach, usually up to 10–12 cm proximal to the wrist crease. First step is carpal tunnel reopening and delicate exploration of the thenar branch as the donor branch for the flap by neurolysis of the median nerve from proximal to distal. If the careful assessment shows this branch not to be viable as donor branch (hidden in scarring, not identifiable from the main median nerve) the first ulnar nerve branch to the abductor digiti minimi (ADM) muscle can be chosen and carefully prepared via opening of the Guyon’s canal. For later muscle insertion the aponeurosis of the abductor pollicis muscle at the base of the thumb proximal phalanx is dissected. As counter-insertion, the dissected ulnar portion of the retinaculum flexorum is freed and prepared. For free flap anastomosis the radial artery on tabatière level or the ulnar artery on wrist level as well as their concomitant veins are dissected. Afterwards the pronator quadratus muscle is prepared underneath the flexor tendons. This allows for maximum length of the neurovascular pedicle [5]. The PQ is approached radially between the deep flexors and the flexor pollicis tendon. First, its proximal border and the entry of the neurovascular bundle (anterior interossea artery, vein and nerve (AIN)) is identified and carefully dissected from the interosseous membrane. The pedicle is followed as proximal as possible, small branches are clipped or ligated and the pedicle it dissected approximately 6 cm proximal to its muscle entry. Then, the whole muscle is carefully released from the radius, the interosseous membrane and from the ulnar attachments. Care is taken not to injure the neurovascular pedicle, which runs on the undersurface of the muscle. Since the nerve coaptation will be located underneath the muscle, the microsurgical part has to be done first. The muscle insertion follows after completion of this part. In our cases, we performed either an end-to-side anastomosis to the radial artery itself or an end-to-end anastomosis to one of the palmar wrist branches. The course of the pedicle is usually nicely protected by the PQ-muscle. At least one, ideally two, vena comitans, are anastomosed. We recommend performing a hand-suture and not to use a coupler system, since this could be palpable through the skin. The AIN is neurorrhaphied to the proximal thenar branch of the median nerve. In case of a very proximal thenar branch damage, but viable proximal stump, a nerve graft (e.g. medial anterior antebrachial nerve) can be interposed between the AIN and the thenar branch. We recommend protection of the anastomosis/neurorrhaphy via fibrin glue embedding and simple interrupted suture wound closure.

In case that the thenar nerve branch is not viable, the vessels have to be anastomosed end-to-side to the ulnar artery and end-to-end to its comitant veins. The AIN than can be easily coapted to the first ADM-branch of the ulnar nerve without tension. Since the ADM usually has at least two branches, there should not be any donor morbidity in terms of muscle force of small digit abduction [13].

**Fig. 1:**
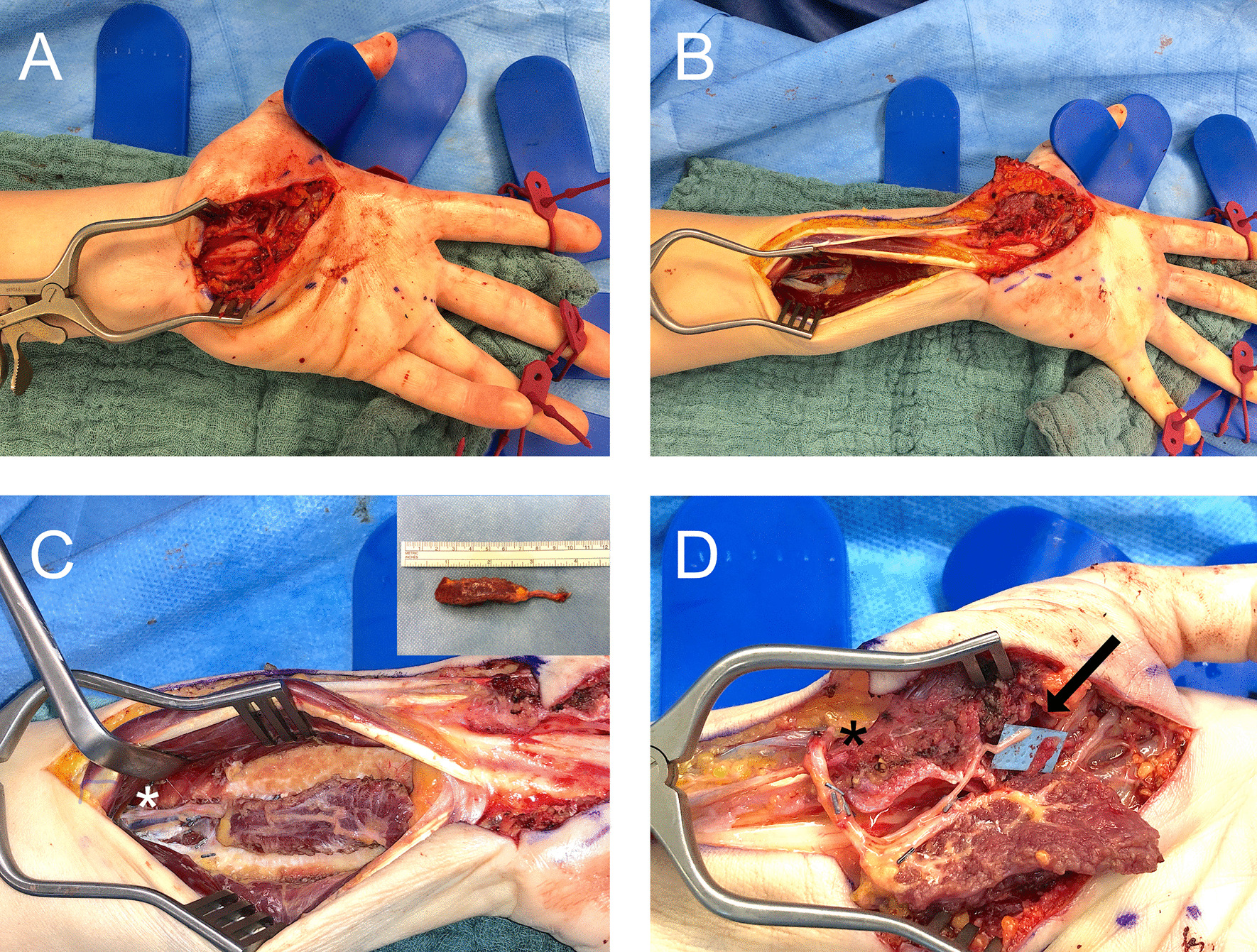
**A** Skin incision and exploration of the thenar branch. **B** To prepare the FFPQ, the incision is prolonged to the distal third of the forearm. **C** The PQ is prepared. * marks the neurovascular bundle of the muscle. **D** The FFPQ is coopted to the thenar branch with the AIN (Arrow) and blood supply is ensured with end-to-side anastomosis to the radial artery. The inlay of the muscle is then sutured to transversal carpal ligament and radially to the abductor aponeurosis

The radial and ulnar muscle borders then have to inserted into their new bed and fixed with PDS 3/0 in the abductor aponeurosis and the ulnar retinaculum border. Care is taken to reach the right pretension of the muscle – after insertion it has to have the same width like it had in situ in the forearm. (Fig. 1).

### Postoperative treatment

Postoperatively a dorsal splint with thumb abduction stop is modulated. We recommend monitoring of the hand for five days for hematoma and compartment syndrome. Flap monitoring can be performed by handheld doppler.

After reduction of the swelling, a brace is fitted with neutral position (30°abduction, 30° opposition) of the thumb to prevent rupture of the muscle inset. Passive physiotherapy can start from the second postoperative day on, with the limitation of thumb movement to a neutral position for six weeks. After six weeks, physiotherapy can start with passive motion further than neutral to regain the range of motion. Depending on the regeneration distance of the nerve usually first contractions of the reinnervated muscle can be observed after 3 months in clinical and neurophysiological examination. With successful reinnervation, regeneration of force should be pursued with consistent training.

**Fig. 2:**
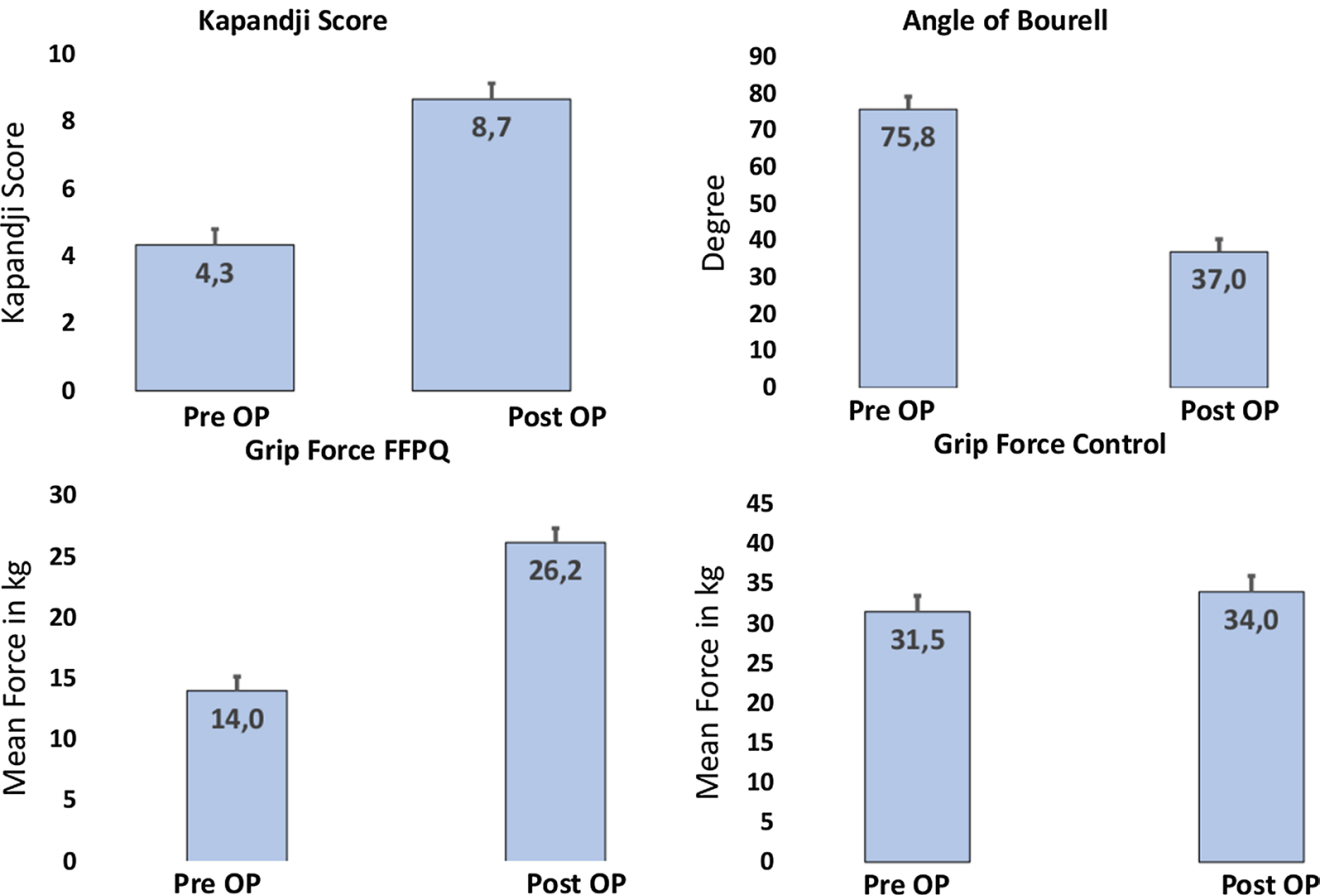
Algorithm and general considerations of thenar reconstruction using the FFPQ

We endorse follow up examinations of the patient after 3, 6, and 12 months. (Fig. 2).

## Results

All flaps were reinnervated successfully and showed excellent regeneration. No revision surgery was needed.

Preoperative Kapandji score for thumb opposition showed a mean of 4.3 ± 0.94. In the two year follow up, thumb opposition regenerated to Kapandji 8.7 ± 0.47. This difference was found to be clinically significant.

Bourrel’s angle between the tip of the thumb and the fourth digit decreased from 75.75 ± 3.45 degrees to 36.96 ± 3.68 degree.

Mean grip strength of the injured hand increased from 14 ± 2.2 kg to 26.2 ± 1.2 kg. Strength measured on the contralateral (uninjured) hand showed a mean force of 31.5 ± 1.5 kg preoperatively and 34 ± 2 kg postoperatively in the 2-year follow up.

**Fig. 3:**
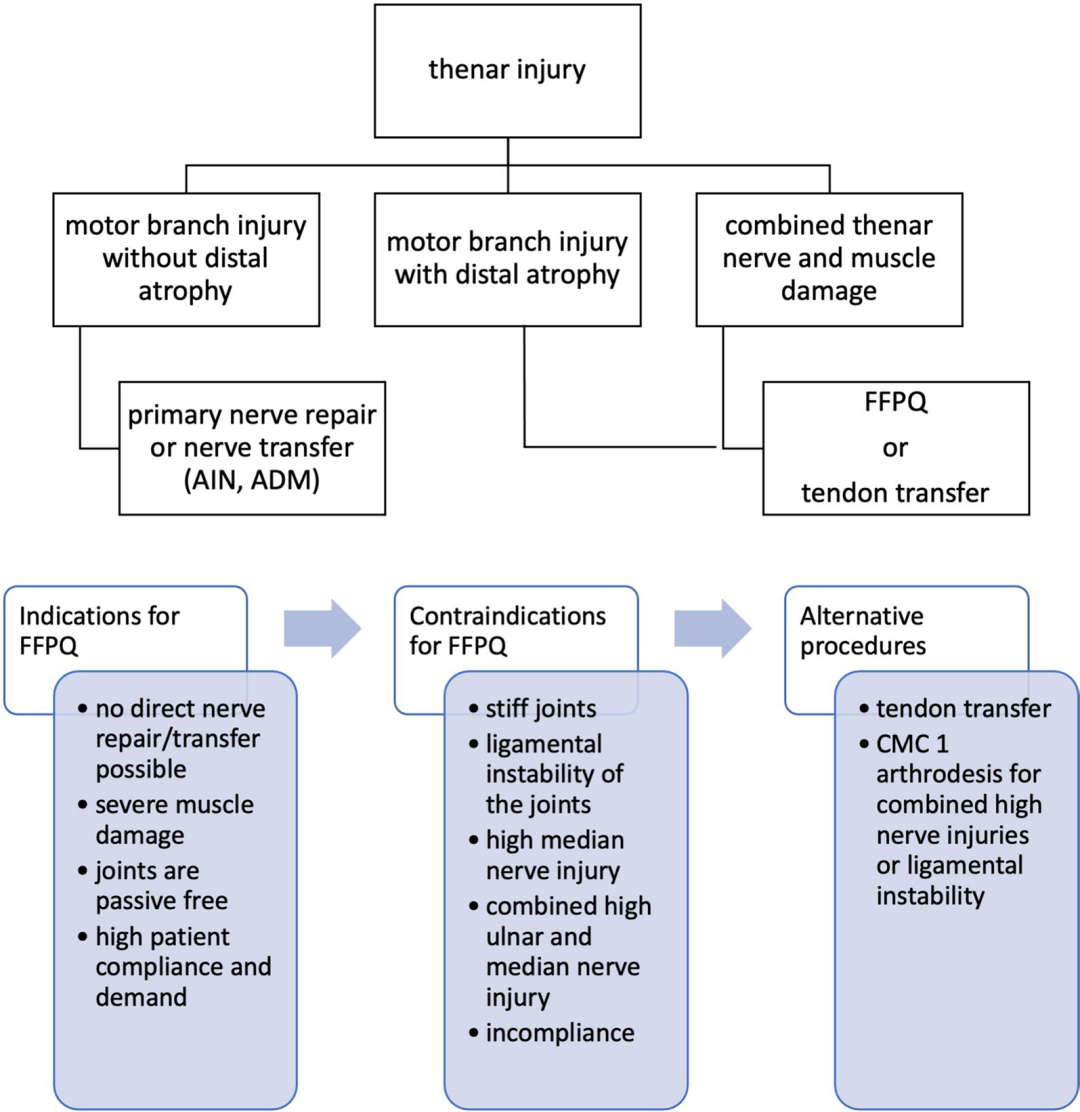
Preoperative Kapandji score showed a mean of 4.3 ± 0.94, which regenerated to Kapandji 8.7 ± 0.47 in the two year follow up. Bourrel’s angle between the tip of the thumb and the fourth digit decreased from 75.75 ± 3.45 degrees to 36.96 ± 3.68 degree. Mean grip strength of the injured hand increased from 14 ± 2.2 kg to 26.2 ± 1.2 kg. Contralateral (uninjured) hand showed a mean force of 31.5 ± 1.5 kg preoperatively and 34 ± 2 kg postoperatively in the two-year follow up

Nerve conduction velocity of the thenar branch was found to be 53.35 ± 1.15 m/s on the uninjured side vs. 46.9 ± 3.1 m/s for the injured side in the follow up (coming from 0 due to completely dissected nerve branch). (Fig. 3).

## Discussion

Traumatic loss of thenar muscle function is a rare injury, that often leads to severe impairment of hand function.

The abductor pollicis brevis (APB) muscle is, besides the opponens pollicis (OP) muscle, the prime muscle of thumb opposition. Therefore, damage to the thenar branch or the muscles itself leads to impairment of opposition rather than a complete loss [14].

Opposition movement of the thumb is the crucial function which makes the human hand unique. Accordingly, impairment is not only disastrous for accomplishing daily life activities and occupational tasks, sometimes leading to psychological burden, but also has socioeconomic consequences [15].

To reconstruct opposition, standardly tendon transfers are used. In 1917, Steindler performed the first opponensplasty using a radial strip of the flexor pollicis longus tendon (FPL) [2]. Subsequently, Huber described the transfer of the abductor digiti minimi in 1921 [16]. Further on, Bunnel (1924) and Camitz (1929) used the palmaris longus muscle with a split tendon transfer to reconstruct opposition [17]. Another viable tendon transfer was described by Caplan et al. in 1956 using the extensor indices muscle and thereby also addressing combined median and ulnar nerve injuries [18].

To broaden the reconstructive repertoire, the first free functional muscle transfer was described in a canine model in 1970 by Tamai et al. [19] and was first successfully used in clinical routine in 1976 [20]. With emerging free flap routine and advancements in microsurgery further viable functional flaps were described.

Accordingly, Dellon et al. were first to describe the PQ as muscle flap of the forearm [5]. Primarily described as a pedicled flap, Lee and Idler [6] and Tzou and Aszmann [7] published case reports using the PQ as a free functional muscle transfer.

**Fig. 4:**
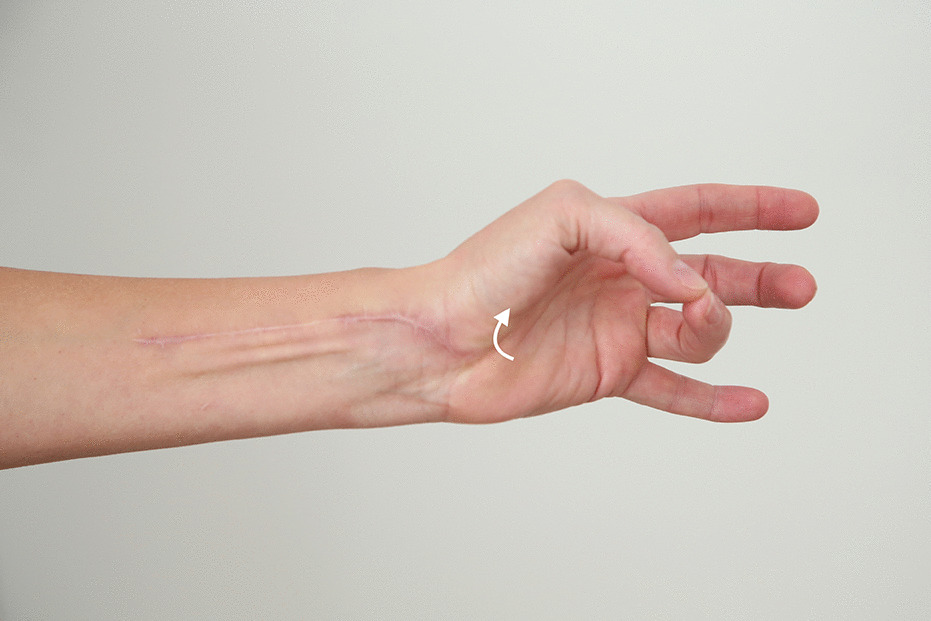
Postoperative outcome 1 year after surgery. Excellent visible and palpable opposition can be observed besides the decent scar of the single incision approach

Without the need for a second incision for flap raising and therefore minimal donor site morbidity, we used the PQ as a FFMT in the largest series of cases so far. Because the original thenar motor branch was used to innervate the flap, no cortical reeducation was necessary for the patients to oppose their thumbs, which was easily feasible for all patients. No impairment in forearm pronation was observed, due to intact pronator teres-function. Other FFMT found in the literature used the serratus flap or a gracilis flap for functional thenar reconstruction [3, 4]. Hereby, the donor site morbidity should be considered higher as with the PQ flap due to the need of a second incision on a different donor site on the body and potential intraoperative relocation of the patient. Furthermore, some of these FFMTs are bulky for delicate hand reconstruction. (Fig. 4).

We found a regeneration of conduction velocity of 53.35 ± 1.15 m/s on the uninjured side compared to 46.9 ± 3.1 m/s to the healthy side with a corresponding recovery of grip strength of 26.2 ± 1.2 kg. Although the force almost doubled compared to preoperative grip force of 14 ± 2.2 kg, there is still a discrepancy to the 34 ± 2 kg on the healthy side. A recent study from Thora et al. [21] showed a mean postoperative grip force of 16 kg after tendon transfer opposition-reconstruction. Although limited patient numbers, this data indicates a superior grip strength after FFMT compared to tendon transfers.

This might be explained with studies from Brand et al. who demonstrated that the PQ has better muscle properties compared to the original thenar muscles considering relative tension and excursion than all described tendon transfers [8, 9].

Bertelli et al. [10] found 649 ± 237 axons in the thenar branch which is almost equivalent to 600 ± 250 axons found in the AIN by Schenk et al. [22]. Furthermore, the number of fascicles (2.3 ± 0.6 for the thenar branch vs. 2.3 ± 1.5 for the AIN) was found to be equal, providing ideal preconditions for regeneration after reinnervation of the thenar branch to the AIN of the PQ.

Considering functional aspects, we found a clinically significant improvement of thumb opposition indicated by a postoperative Kapandji Score of 8.7 ± 0.47 (preoperative 4.3 ± 0.94) with corresponding decrease of Bourrel’s angle to 36.96 ± 3.68 degrees (preoperative 75.75 ± 3.45 degrees).

In comparison, Al-Quattan [23] and Thora et al. [21] described their functional results for tendon transfers as “good” and “excellent” according to the score of Sundararaj et al. [24]. This classification uses the term “excellent” for an opposition of the thumb to the tip of the fourth or fifth digit [23, 24] which is equivalent to a Kapandji score of 5 respectively 6 [12]. Therefore, our results found after FFMT are, due to limited comparability, at least equal to those found after tendon transfer.

**Fig. 5:**
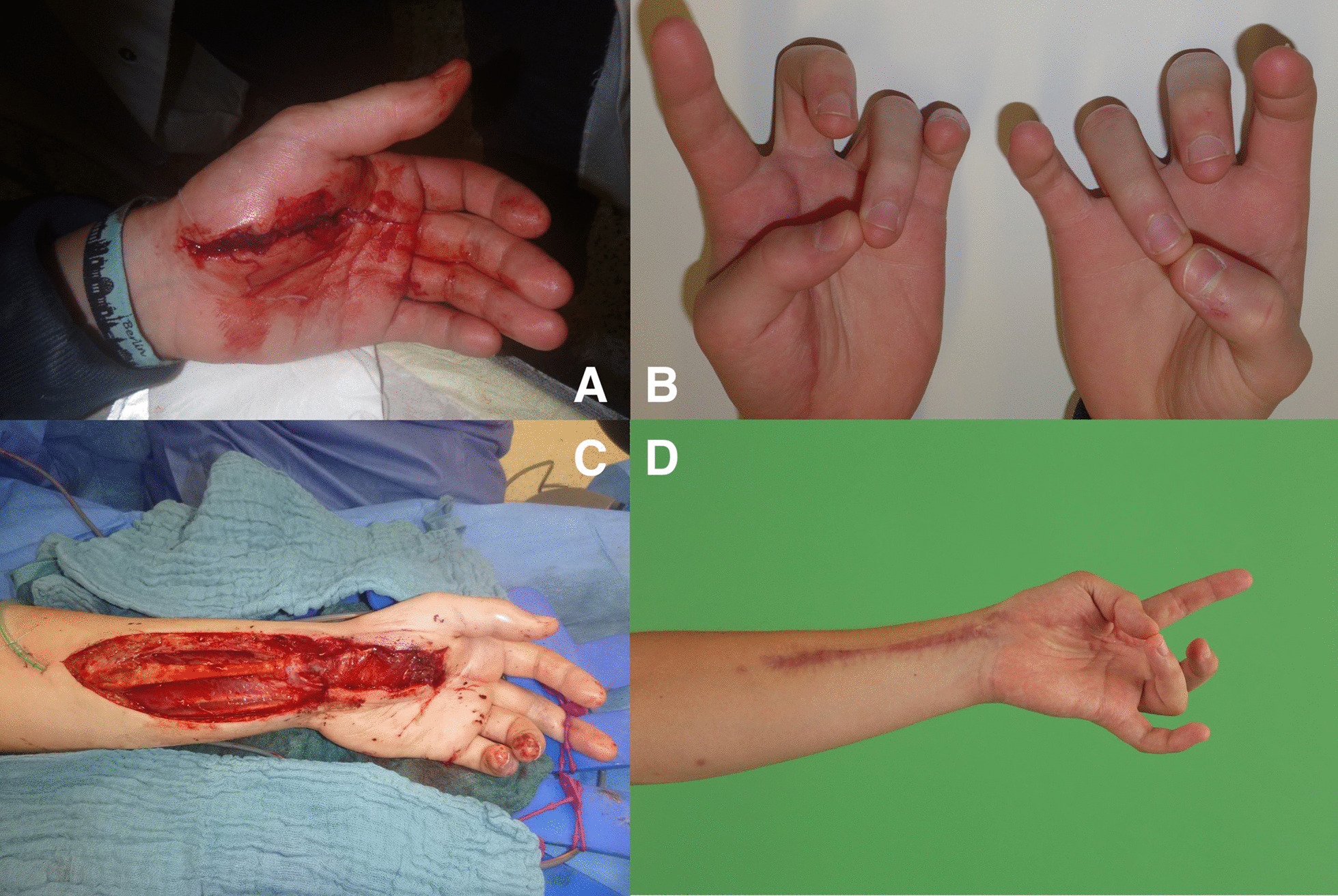
Demaonstartion of a case where we used the FFPQ to reconstruct thumb opposition. **A** The patient suffered from a laceration wound to the thenar branch. **B** After initial wound closure a consequent loss of opposition was observed due to the irreversible damage to the motor entry point of the nerve. **C** Intraoperative setting of the FFPQ using the single incision concept. **D** Functional outcome 6 months postoperatively

Injuries to the thenar muscle mass or motor entry point combined with high demanding patients are excellent indications for a FFPQ. Furthermore, thenar motor branch injuries and prolonged diagnosis with consecutive irreversible muscle damage can be reconstructed with a FFPQ. Due to the single incision procedure, intraoperative exploration of the thenar can then be extended by preparation and transfer of the FFPQ without the need of additional operation sites. Hereby compliance of the patient is as important as free joints to provide sufficient range of motion after regeneration. Still tendon transfers also require compliance and consecutive physiotherapy with the addition of cortical reeducation (Fig. 5).

The FFPQ should not be performed in high median nerve injuries with potential damage to the AIN or PQ. Also combined median and ulnar nerve injuries cannot be reconstructed using the FFPQ due to the absence of a donor nerve. In this scenario, or with ligamental instability of the thumb, a CMC 1 arthrodesis might be a better solution.

## Conclusion

Although injuries to the thenar motor branch or the muscles are rare, they can cause severe impairment of hand function. Hereby tendon transfers are the current standard for reconstruction of thumb opposition. In our study we demonstrate an alternative to tendon transfers with the free functional pronator quadratus flap. Especially young and demanding patients benefit from this procedure with excellent functional recovery of thumb opposition and grip strength. Due to minimal donor site morbidity and the superior benefit of a single incision surgery this flap is highly advantageous compared to other functional free flaps.

Although, the rare indication of thenar reconstruction results in low patient numbers, future studies should further reveal the high potential of the free functional pronator quadratus flap for thenar reconstruction.

## Supplementary Information

**Additional file 1. **FFPQ_outcome_6MoPostOP.

## Data Availability

The datasets used and/or analysed during the current study available from the corresponding author on reasonable request.

## Abbreviations

AIN: Anterior interosseus nerve
ADM: Adductor digiti minimi muscle
APB: Abductor pollicis brevis muscle
FFMT: Free functional muscle transfer
FFPQ: Free functional pronator quadratus
OP: Opponens pollicis
PQ: Pronator quadratus
